# Lack of Consensus Between Measurements of Plasma Phylloquinone by Enzyme-Linked Immunosorbent assays and a Well-Validated High-Performance Liquid Chromatographic Method

**DOI:** 10.1016/j.cdnut.2023.101959

**Published:** 2023-06-03

**Authors:** Rebecca Ruditser, Xueyan Fu, Sarah L. Booth, Minying Liu, Xiaohua Shen, M Kyla Shea

**Affiliations:** United States Department of Agriculture Human Nutrition Research Center on Aging, Tufts University, Boston, MA, United States

**Keywords:** phylloquinone, immunoassay, ELISA, quality assurance, vitamin K

## Abstract

Enzyme-linked i2mmunosorbent assays (ELISAs) that measure circulating phylloquinone have become commercially available, but their validity is uncertain. The objective of this study was to compare plasma phylloquinone concentrations measured using two commercially available ELISAs with concentrations measured using a validated high-performance liquid chromatography (HPLC) assay in 108 samples obtained from participants in a depletion (∼10 mcg phylloquinone/d)–supplementation (∼500 mcg phylloquinone/d) study. The geometric mean of plasma phylloquinone measured with ELISA A was 0.70 nmol/L, 37% lower than that measured with HPLC. The mean of the ELISA B measures was 12.4 nmol/L, >700% higher than the HPLC measures. Plasma phylloquinone measured using HPLC was significantly lower during phylloquinone depletion than supplementation (0.4 ± 0.1 compared with 1.2 ± 0.2 nmol/L; *P* < 0.001). Neither of the two ELISAs detected any significant difference in plasma phylloquinone concentrations between depletion and supplementation (ELISA A, *P* = 0.76; ELISA B, *P* = 0.29). These findings reinforce the need to validate plasma phylloquinone assays as they become available. *Curr Dev Nutr* 2023;x:xx.

## Introduction

The primary circulating form of vitamin K is phylloquinone, and circulating phylloquinone is a biomarker of vitamin K status for research and clinical purposes [1]. The United Kingdom National External Quality Assessment Service is a nonprofit organization consisting of over 20 external quality assessment (EQA) centers across all pathology disciplines. The vitamin K external quality assurance scheme (KEQAS) is one of the EQA affiliated schemes. The aim of KEQAS is to ensure the analytical validity of circulating phylloquinone assays [2] by distributing human serum samples to participating laboratories 4 times per year for the measurement of phylloquinone. Since 1996, over 100 laboratories have participated in KEQAS to confirm the validity of their circulating phylloquinone assays. Although KEQAS uses serum samples, circulating phylloquinone can be measured in both plasma and serum.

Recently, ELISAs to quantify circulating phylloquinone have become commercially available. However, studies comparing ELISA and HPLC circulating phylloquinone measurements are lacking. Therefore, the purpose of this study was to compare circulating phylloquinone measured using two commercially available ELISAs with the concentrations measured using a KEQAS-validated HPLC assay.

## Methods

### Samples

A total of 108 archived plasma samples were obtained from 9 participants in a dietary vitamin K depletion and supplementation crossover study [3]. We selected plasma for the purpose of this study due to availability of samples. During the dietary vitamin K depletion phase, participants consumed a diet that contained ∼10 mcg phylloquinone/d, whereas during the vitamin K supplementation phase, participants consumed the same diet but received a phylloquinone supplement that contained ∼500 mcg phylloquinone/d. Each participant provided between 2 and 22 plasma samples in both fasting and nonfasting states that were used in the present analyses. The protocol was approved by the Tufts University Health Sciences institutional review board [3].

### Laboratory analyses

All analyses were conducted in the vitamin K laboratory at USDA Human Nutrition Research Center on Aging at Tufts University.

#### HPLC

Two hundred microliters of plasma were aliquoted per sample. An internal standard, K_1(25)_ (GL Synthesis), ethanol, distilled water, and hexane were added to each sample. After vortexing for 3.5 min, the samples were centrifuged at 4°C for 5 min at 1107 g. The supernatant was then extracted to a glass tube and dried under nitrogen gas (Organomation Multivap Nitrogen Evaporator) with heat (60°C). Solid phase extraction (SPE) method was used to purify and remove endogenous compounds from the samples. The SPE procedure utilized a Vac-Elute SPS 24 manifold rack with silica SPE columns (Agilent Bond Elut, 500 mg). Columns were conditioned with hexane, and samples were collected in 3.5% ethyl ether in hexane. Samples were dried under nitrogen gas with heat, as previously stated. Samples were subsequently reconstituted in methylene chloride and methanol.

Reverse-phase HPLC included an Agilent series 1260 Infinity II LC System (Agilent Technologies). The HPLC method separates compounds on the basis of reverse-phase separation, using a C18 column (Agilent InfinityLab Poroshell 120 EC-C18; 2.1 × 50 mm, 1.9 μm). Fluorescence detection of phylloquinone was achieved through a reduction reaction utilizing a zinc column (Alfa Aesar Inc). Mobile phase solvent A was 100% methylene chloride. Mobile phase solvent B was methanol and acetonitrile (1:1 vol:vol), 0.55% aqueous solution (2.0 M zinc chloride, 1.0 M acetic acid, and 1.0 M sodium acetate), and 0.45% water. A linear gradient was analyzed as follows: 0% solvent A at 0 min to 10% solvent A at 5.5 min, with flow rate 0.6 mL/min. The mobile phase composition was changed to 30:70 (A:B) at 5.52 min. At 7.51 min, solvent B was increased to 100%. The cycle was finished in 10 min. Response factor (RF) was calculated to measure phylloquinone concentration using the internal standard K_1(25)_. Calibration and sample RF were defined as the phylloquinone peak area divided by the peak area of the internal standard, as quantified in a previously published study [4]. HPLC assay’s lower limit of detection was 0.1 nmol/L. The assay is routinely validated via KEQAS participation [2,5]. The circulating phylloquinone concentrations obtained with this assay are within the acceptable range of KEQAS’s expected values (analyses occurs every 4 mo, for >30 cycles of verification). The intra- and interassay CVs are 4.2% and 4.9%, respectively. HPLC with fluorescence detection for quantification of phylloquinone concentrations has been used in several clinical and pharmacological studies. This, among other HPLC methods, remains in wide use for measuring phylloquinone concentrations in human plasma samples [6].

#### ELISA

Plasma phylloquinone concentrations were also measured using 2 commercially available competitive binding ELISAs: ELISA A (Abbexa, Cambridge, UK) and ELISA B (LSBio, Seattle WA, USA). Both ELISA assays are based on competitive inhibition. The wells of the 96-well plate were precoated with a target-specific antibody. Competitive inhibition occurred between the phylloquinone contained in the plasma samples and the biotinylated phylloquinone conjugate. Unbound antigens were washed away with wash buffer at this step and after incubation with an HRP-conjugated reagent. Per the manufacturer, the reported detection range of ELISA A is 0.0156–1.0 ng/mL (0.0346–2.22 nmol/L), with a sensitivity of 0.00938 ng/mL (0.02063 nmol/L) and intra- and interassay CVs of <10% [7]. Per the manufacturer, the reported detection range of ELISA B is 0.156–10 ng/mL (0.3463–22.2 nmol/L), with a sensitivity of 0.05 ng/mL (0.11 nmol/L), intra-assay CV of <5.1%, and interassay CV of <7.3% [8]. The plasma phylloquinone concentrations were quantified using given standards by GraphPad Prism Version 9.4.

### Statistical analysis

For purposes of statistical analyses, plasma phylloquinone concentrations were converted to nmol/L by multiplying ng/mL by 2.22. The HPLC and ELISA A measurements were natural logarithm (ln) transformed prior to formal analysis to normalize their distributions. (ELISA B measurements had a Gaussian distribution, so no transformation was applied.) Pearson correlation coefficients of ln (ELISA A) and ELISA B measurements with ln (HPLC) measurements were calculated. Bland-Altman plots were generated to identify bias and 95% limits of agreement between each ELISA and the HPLC assay. The response of each assay to phylloquinone supplementation was evaluated using repeated measures ANOVA (SAS proc mixed). For this analysis, we only included samples obtained in a fasting state. This left 34 samples in the depletion phase and 23 samples in the supplementation phase [3]. All statistical analyses were conducted using SAS software package version 9.4.

## Results

**FIGURE 1:**
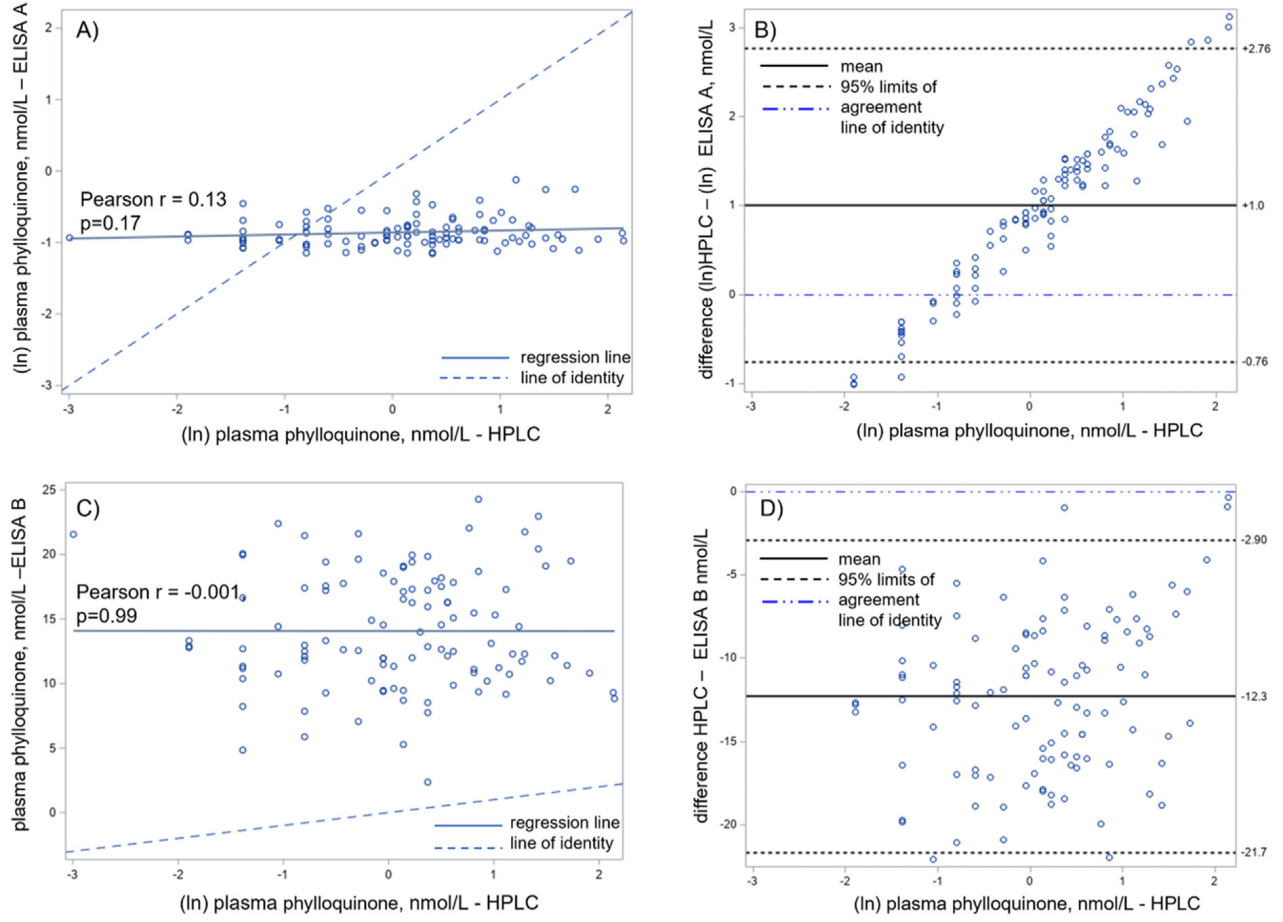
Correlation (A and C) and Bland-Altman (B and D) plots of ELISA A and HPLC measurements (A and B) and ELISA B and HPLC measurements (C and D). ln, natural logarithm.

The overall mean ± SD plasma phylloquinone concentrations were as follows: HPLC, 1.6 ± 1.6 nmol/L; ELISA A, 0.4 ± 0.1 nmol/L; and ELISA B, 14.1 ± 4.5 nmol/L (all *n* = 108). Neither ln (ELISA A) measures nor ln (ELISA B) measures were correlated with ln (HPLC) measures [Pearson *r* = 0.13 (*P* = 0.17) and −0.01 (*P* = 0.99), respectively] (Figure 1A, C).

The geometric mean of ELISA A measures was 0.70 nmol/L, 37% lower than that of HPLC measures [mean difference = ln (HPLC) − ln (ELISA A) = 1.0 nmol/L; 95% limits of agreement: −0.76, 2.76]. The mean of ELISA B measures was 12.4 nmol/L, >700% higher than that of HPLC measures (95% limits of agreement: 2.9, 27.7) (Figure 1B, D).

**FIGURE 2:**
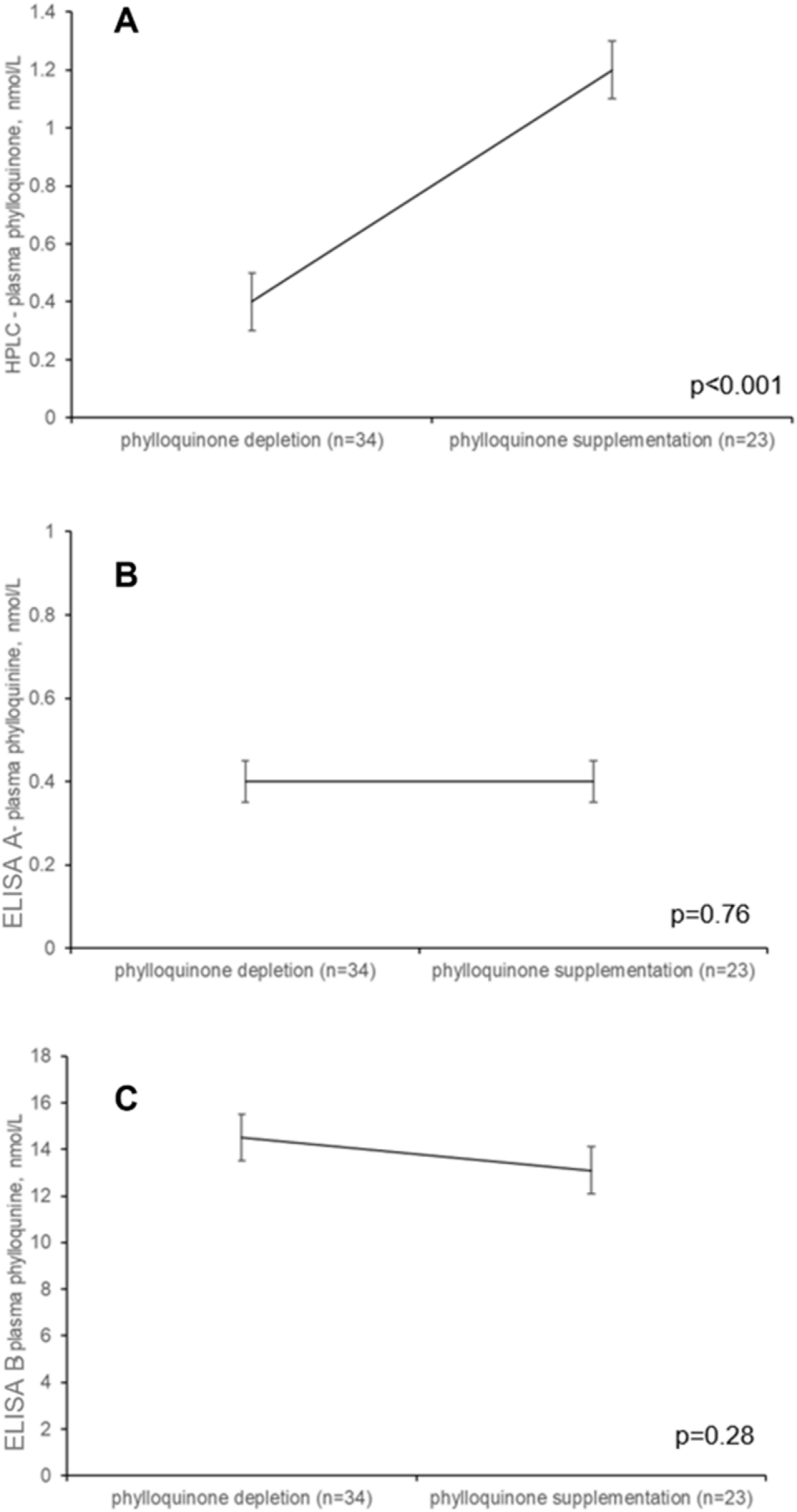
Plasma phylloquinone concentrations measured during dietary phylloquinone depletion and supplementation using (A) HPLC, (B) ELISA A, and (C) ELISA B.

The plasma phylloquinone concentrations measured during dietary phylloquinone depletion and supplementation are shown in Figure 2. Plasma phylloquinone measured using HPLC increased in response to phylloquinone supplementation (Figure 2A). However, the ELISAs did not detect any significant difference in plasma phylloquinone concentrations between the depletion and supplementation phases (Figure 2B, C).

## Discussion

In this study, we demonstrated significant differences in plasma phylloquinone concentrations measured using 2 commercially available ELISA kits compared with measurements obtained using a validated HPLC assay. Compared with HPLC, one ELISA method had consistently lower plasma phylloquinone concentrations, whereas the other had consistently higher plasma phylloquinone concentrations. The use of ELISAs to measure plasma phylloquinone does not yet appear to be widespread, as we did not find any published papers that have utilized these methods. However, given their availability, those interested in measuring circulating phylloquinone using commercially available ELISAs should proceed cautiously until these assays are validated using KEQAS.

In the parent study, plasma phylloquinone measured using HPLC decreased in response to dietary phylloquinone depletion (*P* < 0.001) and increased in response to phylloquinone supplementation (*P* < 0.001) [3]. These findings were consistent with use of multiple measures of vitamin K status, all of which demonstrated a decrease in vitamin K status in response to depletion and subsequent increase in vitamin K status in response to supplementation [3]. Our analysis of plasma phylloquinone using HPLC for the present study confirmed this expected response. However, plasma phylloquinone concentrations measured using ELISA A did not differ between the depletion and supplementation phases, whereas ELISA B detected a slight decrease (albeit nonsignificant) in plasma phylloquinone from the depletion phase to the supplementation phase. The observation that neither of the two ELISAs detected an increase in plasma phylloquinone following phylloquinone supplementation provides additional evidence that these two commercially available ELISAs do not reliably measure plasma phylloquinone.

Parallel analytical challenges have been reported for measurement of other fat-soluble vitamins. For example, He et al. [9] compared 3 commercially available ELISA kits measuring plasma 25(OH)D with LC-MS/MS. The study demonstrated that all 3 ELISAs measuring plasma 25(OH)D had poor agreement and negative bias compared with concentrations measured using LC-MS/MS, which resulted in misclassification of participants’ vitamin D status based on the ELISA measurements [9]. Lai et al. [10] also found that intra- and inter-25(OH)D assay variability resulted in misclassification of vitamin D deficiency, which has clinical implications. Circulating phylloquinone is measured clinically in the context of intestinal malabsorption, liver disease, or inborn errors of metabolism as well as for research purposes. Hence, misclassification can be problematic in clinical and research settings.

In conclusion, our study demonstrated that plasma phylloquinone concentrations measured using 2 commercially available ELISAs were not consistent with the measurements obtained using a validated HPLC assay. Until these ELISAs are rigorously validated, circulating phylloquinone concentrations should be measured using a validated HPLC or LC-MS assay.

## Funding

Supported by the Unites States Department of Agriculture Agricultural Research Service Cooperative Agreement 58-8050-9-004.

## Author disclosure

KS is an Academic Editor for Current Developments in Nutrition. She was not involved in the journal’s evaluation of this manuscript. RR, XF, SLB, ML, XS, no conflicts of interest.

## Author contributions

The authors’ responsibilities were as follows—RR, XF, and MKS: designed the study; RR, XF, and XS: conducted the laboratory analyses; MKS: analyzed the data; RR, XF, SLB, ML, and MKS: wrote the manuscript; MKS and SLB: responsible for final content; and all authors: read and approved the final manuscript.

## Data availability

Data described in the manuscript and analytic code will be made available upon request pending approval of pertinent data sharing agreements.

## References

[1] Shea M.K., Booth S.L. (2016). Concepts and controversies in evaluating vitamin K status in population-based studies. Nutrients.

[2] Card D.J., Shearer M.J., Schurgers L.J., Harrington D.J. (2009). The external quality assurance of phylloquinone (vitamin K (1)) analysis in human serum. Biomed. Chromatogr..

[3] Truong J.T., Fu X., Saltzman E., Al Rajabi A., Dallal G.E., Gundberg C.M. (2012). Age group and sex do not influence responses of vitamin K biomarkers to changes in dietary vitamin K. J. Nutr..

[4] Fu X., Peterson J.W., Hdeib M., Booth S.L., Grusak M.A., Lichtenstein A.H. (2009). Measurement of deuterium-labeled phylloquinone in plasma by high-performance liquid chromatography/mass spectrometry. Anal. Chem..

[5] Card D., Freke E., Harrington D. (2022). Characterization and traceability of two generations of standard reference material for the measurement of vitamin K_1_(phylloquinone) at endogenous concentrations in human plasma and serum. Biomed. Chromatogr..

[6] Zhang Y., Bala V., Mao Z., Chhonker Y.S., Murry D.J. (2019). A concise review of quantification methods for determination of vitamin K in various biological matrices. J. Pharm. Biomed. Anal..

[7] Antibodies, proteins, ELISA kits [Internet]. Abbexa [cited February 15, 2023]. Available from: https://www.abbexa.com/vitamin-k1-vk1-elisa-kit-3.

[8] Phylloquinone (competitive EIA) Elisa Kit - LS-F10661 [Internet]. LSBio [cited February 15, 2023]. Available from: https://www.lsbio.com/elisakits/phylloquinone-competitive-eia-elisa-kit-ls-f10661/10661.

[9] He C.S., Gleeson M., Fraser W.D. (2013).

[10] Lai J.K., Lucas R.M., Banks E., Ponsonby A.L. (2012). Ausimmune Investigator Group, Variability in vitamin D assays impairs clinical assessment of vitamin D status. Intern. Med. J..

